# Relationships between nitrogen cycling microbial community abundance and composition reveal the indirect effect of soil pH on oak decline

**DOI:** 10.1038/s41396-020-00801-0

**Published:** 2020-10-16

**Authors:** K. Scarlett, S. Denman, D. R. Clark, J. Forster, E. Vanguelova, N. Brown, C. Whitby

**Affiliations:** 1 Forest Research, Centre for Forestry and Climate Change, Farnham, GU10 4LH UK; 2 School of Life Sciences, University of Essex, Wivenhoe Park, Colchester, CO4 3SQ UK; 3 Institute for Analytics and Data Science, University of Essex, Wivenhoe Park, Colchester, CO4 3SQ UK

**Keywords:** Biogeochemistry, Soil microbiology, Microbial ecology

## Abstract

Tree decline is a global concern and the primary cause is often unknown. Complex interactions between fluctuations in nitrogen (N) and acidifying compounds have been proposed as factors causing nutrient imbalances and decreasing stress tolerance of oak trees. Microorganisms are crucial in regulating soil N available to plants, yet little is known about the relationships between soil N-cycling and tree health. Here, we combined high-throughput sequencing and qPCR analysis of key nitrification and denitrification genes with soil chemical analyses to characterise ammonia-oxidising bacteria (AOB), archaea (AOA) and denitrifying communities in soils associated with symptomatic (declining) and asymptomatic (apparently healthy) oak trees (*Quercus robur* and *Q. petraea*) in the United Kingdom. Asymptomatic trees were associated with a higher abundance of AOB that is driven positively by soil pH. No relationship was found between AOA abundance and tree health. However, AOA abundance was driven by lower concentrations of NH_4_^+^, further supporting the idea of AOA favouring lower soil NH_4_^+^ concentrations. Denitrifier abundance was influenced primarily by soil C:N ratio, and correlations with AOB regardless of tree health. These findings indicate that amelioration of soil acidification by balancing C:N may affect AOB abundance driving N transformations, reducing stress on declining oak trees.

## Introduction

Tree health is a global concern as they provide essential ecosystem services, yet significant areas of forests are being lost due to pest and disease outbreaks or environmental change. In temperate regions, there is an increase in oak tree decline and mortality rates [1–3]. Several biotic agents and abiotic stressors have been proposed as contributing factors to oak decline, including increased temperatures, pollution, invasive pests and pathogens [4–9]. Due to the complex interactions that occur between these stressors and agents, identification and amelioration of soil stressors that lead to decline symptoms is a challenge in oak health management [10–14].

Across the United Kingdom and Europe, many oak trees are experiencing an increase in nitrogen (N) deposition and acidifying compounds, which have been proposed as factors destabilising the circulation of nutrients and reducing stress tolerance [4, 15]. In N-limited systems, an increase in soil N has been suggested to encourage vegetative growth [16] that increases the susceptibility of trees to damage by insects and plant pathogens [15, 17] thereby changing insect activity and insect population densities [18] and decreasing frost hardiness of trees [19]. In contrast, soils deficient in N may inhibit plant growth, resulting in weaker and slower growing plants, that may be more susceptible to insect attack (e.g. *Agrilus*) and pathogens [16, 20, 21]. Thus, disentangling the direct and indirect effects of soil N availability on tree health is difficult, largely due to the confounding influences of plant pathogens and insect pests, plant physiology, root microclimate and the soil microbiome [20, 22].

Microorganisms play a pivotal role in soil N-cycling and regulating soil N available to plants [23]. Two key processes (i.e., nitrification and denitrification), are especially important in this context. The critical process, and rate-limiting step, of autotrophic nitrification involves the oxidation of ammonium (NH_4_^+^) to nitrite (NO_2_^−^) and is driven by ammonia-oxidising bacteria (AOB) and archaea (AOA) [24, 25]. Nitrite is subsequently oxidised to nitrate (NO_3_^−^), resulting in a net loss of N from the ecosystem, via leaching, and/or denitrification [24, 25].

Although AOA and AOB co-exist in soils, they respond differently to soil environmental factors and there is evidence of niche differentiation between AOB and AOA [25–27]. For example, the global dominance of AOA in acidic soils [24, 25, 28], and AOA rather than AOB favouring low ammonium environments, like unfertilised soils [29–34]. Additionally, studies have found plant modulation of AOA and AOB community abundance, whereby a higher abundance of AOB has been found under plant canopies, possibly due to higher N concentrations that results from an increase in plant litter [35, 36]. Changes in the microbial utilisation of organic carbon and nitrogen can also alter the overall soil microbial community structure [37–39], which in turn may impact nitrification. In order to determine if destabilization of soil N-cycling is associated with oak stress and decline, a better understanding of the mechanisms that control the abundance and diversity of soil microbes that mediate these N-transformation processes is required.

In this study we characterised the abundance and diversity of the microbial communities driving the N transformations in soil associated with symptomatic and asymptomatic, *Quercus robur* and/or *Q. petraea* trees across seven woodlands in the UK. We used piecewise structural equation modelling to analyse the direct and indirect effects of the soil abiotic environment on N-cycle microbial communities in relation to oak tree health. We hypothesised that the structure and abundance of N-cycling microbial communities would differ between symptomatic and asymptomatic trees due to inherent differences in soil chemical properties that may be affecting microbial composition and function.

## Materials and methods

### Sample sites and strategy

**Fig. 1:**
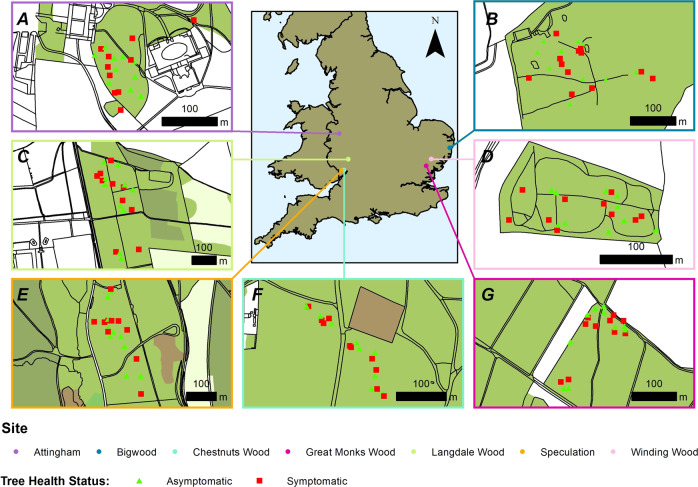
Location of seven woodland sites used in this study, sampled between the years 2016–2017. **A** Attingham; **B** Bigwood; **C** Langdale; **D** Winding Wood; **E** Speculation; **F** Chestnuts Wood; **G** Great Monks Wood. Ten symptomatic (red) and ten asymptomatic (green) trees are indicated at each site).

This study was conducted in seven oak dominated broadleaf woodlands across England, where both declining oak trees (symptomatic) and non-declining (asymptomatic) trees were present (Fig. 1). Ten symptomatic and ten asymptomatic trees were selected at each site based on their phenotypic crown condition [21], as well as either the presence of stem lesions associated with acute oak decline [5], or the presence of stem lesions associated with root decay fungi (*Armillaria* sp. or *Gymnopus fusipes*) (data not shown). Major soil groups were identified at each site using Cranfield University 2020, *The Soils Guide* [40]. Attingham, Bigwood, Great Monks Wood, Langdale and Speculation were identified as surface water gleys, while Chestnuts Wood and Winding Wood were identified as brown soils (Table S1). Oak tree density was variable across the sites ranging from 214.29 oak trees/ha at Chestnuts wood to 30.95 oak trees/ha at Langdale (Table S1). The mean basal area of the studied oak trees was also variable across the sites ranging from 32.55 m^2^/ha at Attingham to 11.55 m^2^/ ha at Bigwood (Table S1). At Attingham, Chestnuts and Winding Wood the mean basal area of the studied oak trees was higher than the recommended threshold of 22 m^2^/ha [41]. Soil samples were taken from the top 0–20 cm of the soil profile, from three evenly spaced soil cores, located 2 m from the base of each tree. Across all the sites sampled, rainfall had occurred either one day prior to, or during, sampling. A total of 420 soil cores were collected across the seven study sites. Soils were placed on ice at 4 °C for 5 h in the field before being stored at −20 °C.

### Soil chemical analysis and nitrification potentials

Soil samples were prepared and analysed for soil moisture content and chemical properties by Forest Research laboratory services at Alice Holt Lodge, Farnham, UK following the EU ICP Forests Soil Manual [42]. Soil moisture content was analysed by oven drying at 105 °C for 24 h or until constant weight was reached. Total C and N concentrations were measured using a C:N Elemental Analyzer, Carlo Erba Flash EA 1112 Series (Thermo Scientific, UK). Soil pH was measured in water suspension (soil:water ratio 1:2.5). Available soil nitrate (NO_3_^−^) and ammonium (NH_4_^+^) were extracted from fresh soil cores using 1 M KCl and measured by colorimeter analysis [43].

Soil nitrification potentials were measured using a shaken soil-slurry as previously described [44] supplemented with NH_4_^+^ (0.3 mM NH_4_Cl). NH_4_^+^ concentration was measured using a Dionex ICS-3000 (Thermo Scientific, UK) as previously described [45]. Rates of NH_4_^+^ removal were determined by linear regression analysis of NH_4_^+^ concentration with time.

### qPCR and amplicon sequencing of N-cycle genes

DNA was extracted from 0.25 g wet weight soil using a Soil DNA Isolation Plus Kit (Norgen Bioteck Corp., Canada) following the manufacturers recommendations. Gene abundance was quantified by qPCR with a SensiFAST SYBR No-ROX Kit (Bioline) on a CFX96 Real-Time PCR Detection System (BioRad) using the following primers: ammonia monooxygenase (*amoA*) genes from archaea—CrenamoA-23F/CrenamoA-616R [46], and bacteria—amoa-1F/amoA-2R [47], *nirS* (nitrite reductase) gene nirsS-Cd3aF/nirS-R3cd [48], *nirK* (nitrite reductase) gene nirK-1F/nirK-5R [49] and *nosZ* (nitrous oxide reductase) gene nosz2F/nosz2R [50].

Thermocycling involved an initial denaturation at 95 °C for 3 min followed by 40 cycles of 95 °C for 5 s, 60 °C for 30 s, which included a combined annealing and extension time. Product specificity was confirmed using melting point analysis (52–95 °C with a plate read every 0.5 °C) and amplicon size was verified with agarose gel electrophoresis. Gene abundances were quantified with an absolute quantification method against an internal standard calibration curve using DNA standards of each target gene from 10^1^ to 10^8^ copies in 20 µl reactions containing 200 nM of primers and 1 µl of DNA template. *R*^2^ values for the standards curves were >0.99 and slope values were between −3.2 and −3.4 giving estimated amplification efficiency between 104 and 95%. Standards, samples and non-template controls were run in duplicate and samples were diluted 10× before adding to the master mix.

Amplicon libraries were prepared by a 28 cycle PCR amplification using Illumina Nextera adapted primers and MyTaq Red DNA polymerase (Bioline, UK). The adapted primers used for gene sequencing included: Bakt 341F/Bakt 805R [51] for 16S rRNA bacteria; 344F/915R [52, 53] for 16S rRNA archaea; as well as *amoA* AOB, *amoA* AOA, *nirS* and *nosZ* nitrogen cycle functional genes (as listed above). Amplicon quality was assessed by visualisation using gel electrophoresis, followed by purification with AMPure XP (Agencourt) SPRI bead protocol and amplification in a further eight cycles of PCR to attach one of 96 unique combinations of Nextera paired-end indexes. After a second round of purification with the AMPure XP beads, the final amplicon was quantified using the Quant-iT Picogreen dsDNA assay kit (Life Technologies) and the Nanodrop 3300 fluorospectrometer (Thermo Scientific) and then pooled in equimolar concentrations. The amplicon libraries were quality checked using a DNA 1000 kit on a 2100 Bioanalyzer (Agilent) before final pooled libraries were sequenced on the Illumina Miseq platform using a MiSeq reagent kit V3 (2 × 300 bp) at The University of Essex (Colchester, Essex, UK).

### Assembly and analysis of sequencing reads

Sequence reads were demultiplexed on the MiSeq platform and analysis was performed as described by Dumbrell et al. [54]. The sequences were quality trimmed using Sickle [55], with a min quality threshold of q20. Sequences were then error corrected with SPAdes [56] using the BayesHammer algorithm [57]. The sequences were then de-replicated, sorted by abundance and OTU centroids were picked using VSEARCH [58] at 97% similarity. Singleton OTUs were removed from the dataset, along with any chimeric sequences identified by both de novo and reference-based chimera checking with UCHIME [59]. Taxonomy assignment for 16S rRNA sequences was performed with the RDP Classifier [60]. For all functional genes (*amoA, nirS, nosZ*), non-locus-specific OTUs were detected and filtered using the online FrameBot tool with default settings [61]. Centroid sequences of the most abundant OTUs (comprising >99% of the sequencing reads for each gene) were aligned by codons using MUSCLE, in the MEGA6 program [62, 63]. Sequences were aligned with other known functional gene sequences from the FunGene database [64] and from BLAST analyses [65].

### Statistical analyses

All analyses were conducted in R Studio (version 2.1; R Development Core Team, 2016). Piecewise structural equation modelling (Piecewise SEM) [66] was used to evaluate the direct and indirect relationships between the soil abiotic variables (soil carbon, pH, NH_4_^+^, NO_2_^−^ + NO_3_^−^, C:N ratio, soil moisture, nitrification potential), N-cycle microbial gene abundances (*amoA* AOA, *amoA* AOB, *nirS*, *nirK*, *nosZ*) and tree health status (asymptomatic or symptomatic). We generated the median value of the three core replicates per tree for use in statistical analyses. Construction of the priori model required checking normality of the endogenous variable and further log-transforming the gene abundance data to improve normality. Tree health status was given a binary assignment (1: asymptomatic; 0: symptomatic) to allow comparisons between this categorical variable. The priori model included the combined effects of all soil abiotic variables and N-cycle genes on tree health status as a composite model. The random effects of each of the seven wood sites were also accounted for in the model. We assessed the fit of the model using Shipley’s test of directed separation, which tests the assumption that all variables are conditionally independent, indicating that there are no missing relationships among unconnected variables, supported by a non-significant Fisher’s C value (*P* > 0.05) [67]. We could then interpret the path coefficients of the model (that describe the strength and direction of the relationship of the variables) and their *P* values [68]. After assessing the overall relationships between the *amoA* AOB and AOA gene abundance with tree health through the piecewise SEM, we constructed a separate quasibinomial generalised linear model (*glm*) and assessed the least squared means to determine the relationship between the ratio of AOB:AOA associated with tree health status at each of the sites.

After discarding excessively small samples, OTU tables were rarefied to an even depth with the “*vegan*” package [69] including 900 sequences per sample for AOB, 800 for AOA, 500 for *nir*S and 700 for *nosZ*. Compositional differences in the soil microbial communities of each gene functional group were quantified using model-based analysis of multivariate abundance data using the mvabund package in R-studio [70] and visualised using non-metric multidimensional scaling (NMDS) (two axes) on the Bray–Curtis distance matrix using the metaMDS function in the vegan package [69]. Raw sequence data were submitted to the European Nucleotide Archive under accession number PRJEB35364.

Phylogenetic analysis was performed using the 50 most abundant OTUs from each of the genes sequenced. Representative OTU gene sequences and reference sequences, from NCBI Blast Database, were aligned using the ClustalW alignment followed by neighbour-joining phylogenetic trees with calculated bootstrap analysis (based on 1000 replicates) using Geneious package 9.0.2 (https://www.geneious.com).

## Results

### Soil chemical characteristics and nitrification potential rates

The soils were highly heterogeneous across and within the seven sites. Across sites, pH ranged from the more acidic soils at Winding Wood (pH 3.6 to up to 7.6), to less acidic soils at Langdale (pH 4.7 to 8.3; Table 1). When comparing NH_4_^+^ concentration between sites, irrespective of tree health status, Great monks wood, Winding Wood, Langdale and Attingham, showed higher NH_4_^+^ concentrations (medians ranging between 11.3 and 29.4 g N kg^−1^ dry soil) compared to Speculation, Chestnuts and Bigwood (1.2–2.5 g N kg^−1^ dry soil). This allowed for two clear groups of sites (1) those with high ammonium (Attingham, Great Monks Wood, Langdale and Winding Wood) and (2) those with lower ammonium concentrations (Bigwood, Chestnuts and Speculation). NO_2_^−^ + NO_3_^−^ concentration at Attingham, Great Monks Wood and Langdale had lower NO_2_^−^ + NO_3_^−^ (1.7, 1.7 and 2.3 g N kg^−1^ dry soil respectively) compared with Bigwood, Chestnuts and Speculation (6.7, 6.8 and 4.1 respectively) (Table 1). Winding Wood was found to have high levels of NO_2_^−^ + NO_3_^−^, particularly in association with asymptomatic trees (23.4 g N kg^−1^ dry soil). While net nitrification potential was generally low across all soils (supporting both symptomatic and asymptomatic trees), the highest nitrification potential rates were found at Langdale (for both asymptomatic and symptomatic); which corresponded to the highest NH_4_^+^ concentration.

**Table 1:** Chemical properties of soil associated with tree health status among different wood study sites (median value, minimum and maximum value).

Health status	Study site	Moisture content (%)	NO_2_^−^ + NO_3_^−^ (g N kg^−1^ dry soil)	NH_4_^+^ (g N kg^−1^ dry soil)	pH	Carbon (g kg^−1^)	C:N ratio	Nitrification potential (μmol NH_3_ g^−1^ per day)
Asymptomatic	Attingham	18.6 (8.4, 62.7)	1.6 (0, 41.0)	16.8 (0, 82.6)	4.7 (3.9, 5.5)	5.6 (2.4, 14.0)	15.6 (12.8, 19.4)	0.12 (0.0, 0.4)
	Bigwood	18.6 (4.5, 36.7)	5.4 (1.4, 13.4)	1.5 (0.3, 27.4)	4.4 (4.0, 7.7)	3.5 (2.3, 6.8)	16.5 (13.3, 22.0)	0.04 (0.0, 0.19)
	Chestnut	23.5 (9.7, 73.3)	7.4 (3.4, 17.5)	2.5 (0.8, 15.4)	4.2 (3.8, 5.2)	2.8 (1.4, 10.2)	15.5 (13.4, 32.4)	0.03 (0.0, 0.13)
	Great monks wood	19.1 (15.1, 40.3)	1.7 (1.4, 30.4)	20.0 (6.3, 53.3)	4.6 (4.1, 7.5)	3.9 (2.3, 18.2)	16.5 (14.0, 19.5)	0.05 (0.0, 0.14)
	Langdale	22.2 (13.1, 37.3)	2.2 (1.4, 6.2)	28.3 (2.2, 40)	5.8 (5.5, 8.3)	4.9 (1.7, 8.6)	13.3 (12.2, 16.0)	0.43 (0.26, 0.61)
	Speculation	26.5 (14.2, 49.0)	4.5 (0.7, 39.0)	1.9 (0.8, 8.15)	4.3 (4.0, 4.7)	9.3 (2.9, 51.1)	24.5 (14.6, 36.7)	0.09 (0.0, 0.16)
	Winding wood	31.4 (11.8, 66.0)	23.4 (1.5, 153.0)	12.4 (3.4, 218)	4.0 (3.6, 7.6)	7.3 (2.5, 34.4)	18.5 (14.6, 21.6)	0.33 (0.13, 0.90)
Symptomatic	Attingham	18.8 (11.6, 45.4)	1.8 (0, 39.9)	16.2 (0, 79.6)	4.5 (4.1, 6.7)	4.1 (2.2, 17.7)	16.1 (14.0, 21.3)	0.13 (0.0, 0.24)
	Bigwood	15.4 (10.4, 26.8)	7.9 (3.1, 17.4)	0.9 (0.2, 19.5)	4.7 (4.1, 7.1)	3.9 (1.3, 5.5)	16.6 (12.3, 23.8)	0.04 (0.02, 0.12)
	Chestnut	26.3 (4.8, 89.2)	6.2 (2.8, 39.4)	2.5 (0.6, 45)	4.2 (3.9, 4.6)	3.0 (1.5, 6.8)	15.4 (13.8, 17.8)	0.04 (0.00, 0.21)
	Great monks wood	19.5 (13.6, 35.9)	1.7 (1.4, 15.7)	15.2 (4.0, 53.3)	4.6 (3.9, 5.2)	3.3 (1.6, 17.6)	16.0 (13.9, 20.9)	0.06 (0.02, 0.11)
	Langdale	24.4 (15.1, 36.5)	2.4 (1.5, 10.2)	30.4 (16.7, 70.4)	5.7 (4.7, 7.5)	4.5 (3.1, 8.3)	13.4 (11.4, 19.1)	0.40 (0.23, 0.59)
	Speculation	29.5 (21.3, 42.7)	3.7 (1.3, 17.6)	1.1 (0.72, 4.9)	4.2 (4.1, 5.7)	6.7 (2.3, 16.4)	19.5 (13.8, 31.1)	0.12 (0.01, 0.41)
	Winding wood	27.6 (10.2, 61.6)	7.1 (1.8, 102.0)	11.3 (3.6, 5.2)	4.1 (3.6, 4.8)	8.3 (3.5, 36.8)	18.6 (16.1, 20.7)	0.28 (0.12, 0.51)

### Relationship between soil parameters and N-cycle genes on tree health

**Fig. 2:**
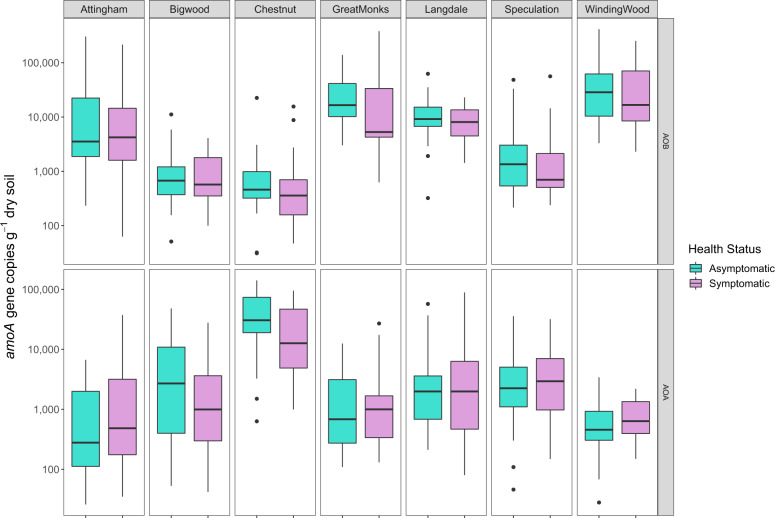
Abundance of AOA and AOB ammonia monooxidase (*amoA*) gene copies in relation to symptomatic and asymptomatic oak trees across seven UK woodlands. Data are medians with upper and lower quartile (*n* = 10), dots are outliers.

The abundance of *amoA* genes for AOA ranged from 2.6 × 10^1^ to 1.4 × 10^5^ gene copies g^−1^ dry weight soil with the highest abundances found at Chestnuts, Bigwood and Speculation, while AOB *amoA* gene abundance ranged from 3.1 × 10^1^ to 4.1 × 10^5^ gene copies g^−1^ dry weight soil with the highest abundances of AOB found at Winding Wood and Great Monks Wood (Fig. 2). Similarly, the abundance of nitrate reductase (*nirS, nirK)* and nitrous oxide reductase (*nosZ)* genes also varied significantly across the sites (*P* < 0.001) (Fig. S1). The abundance of *nirS* genes ranged from 1.4 × 10^3^ to 8.4 × 10^5^ *nirS* gene copies g^−1^ dry weight soil, *nirK* gene ranged from 2.7 × 10^2^ to 8.7 × 10^5^ gene copies g^−1^ dry weight soil and *nosZ* gene abundance ranged from 1.1 × 10^3^ to 2.2 × 10^7^ gene copies g^−1^ dry weight soil (Fig. S1).

**Fig. 3:**
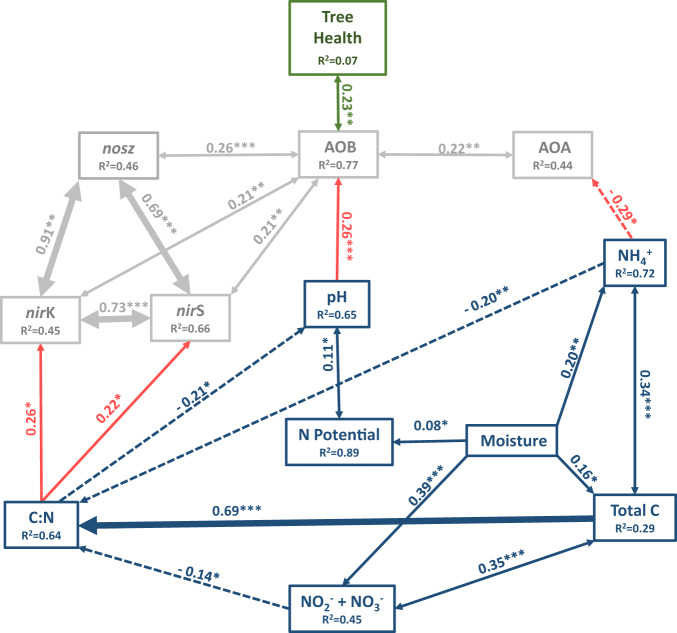
Path diagram for Piecewise structural equation modelling showing only significant direct and indirect effects of soil abiotic variables and N-cycle microbial communities on tree health status. Numbers adjacent to the arrows are standardised path coefficients, analogous to relative regression weights and indicative of the effect size of the relationship. Solid and dashed arrows indicate positive and negative relationships, respectively. Double-headed arrows indicate covariance between variables, single-headed arrows indicate a one way directed relationship. Blue arrows indicate the relationship between soil variables, red arrows indicate the relationships between soil variables and gene abundances, grey arrows indicate the relationships between gene abundances and green arrows indicate the relationship between gene abundances and tree health status. The width of the arrow is proportional to the strength of path coefficients. The proportion of variance explained (conditional *R*^2^) appears below every response variable in the model. Significance levels are as follows: *P < 0.05, **P < 0.01 and ***P < 0.001.

Our a priori Piecewise SEM model provided a satisfactory fit to our data, as suggested by the non-significant Fisher’s C *P* value (*P* = 0.977; df = 8; Fig. 3). The SEM model revealed that asymptomatic trees were associated with a higher abundance of AOB *amoA* gene copies than symptomatic trees (path coeff. = 0.23; *R*^*2*^ = 0.07; *P* = 0.008; Fig. 3; green arrow). In turn, the abundance of AOB *amoA* genes was driven by pH, with increased abundance in higher pH soils (path coeff. = 0.26; *R*^*2*^ = 0.77; *P* < 0.001) (Fig. 3; red arrow) supporting our hypothesis that soil conditions exert an indirect effect on tree health status by modulating ammonia-oxidising bacterial abundance (Fig. 3). In contrast, we observed no association between AOA *amoA* abundance and tree health. AOA abundance instead was primarily driven by soil NH_4_^+^ concentration.

Unexpectedly, the abundance of denitrification genes showed associations with soil parameters and each other, but not tree health status. The C:N ratio proved to be the direct primary driver of nitrite reductase gene abundance (*nirS;* path coeff. = 0.22; *P* = 0.01, *nirK*; path coeff. = 0.26; *P* = 0.02) (Fig. 3; grey arrows). Nitrate reductase genes *nirS* and *nirK* were found to highly positively covary (path coeff. = 0.73; *P* < 0.001) with each other and with nitrous oxide reductase gene (*nosZ*; path coeff. = 0.69; 0.91; *P* < 0.001) abundance (Fig. 3; grey arrows). In addition, AOB was found to positively covary with *nirS* (path coeff. = 0.21; *P* < 0.001)*, nirK* (path coeff. = 0.21; *P* = 0.007) as well as *nosZ* (path coeff. = 0.26; *P* < 0.001) gene abundance (Fig. 3; grey arrows).

Soil parameters were also associated with each other, further demonstrating the complex relationship between soil, microbiomes and tree health. Soil pH was largely influenced by C:N ratio, where a direct negative relationship between NO_2_^−^ + NO_3_^−^ (path coeff. = −0.14; *P* = 0.04) and NH_4_^+^ (path coeff. = −0.20; *P* = 0.003), as well as a positive relationship between total soil carbon (0.69; *P* < 0.001) affected C:N ratio and in turn was found to drive pH (path coeff. = −0.21; *P* < 0.05). Moisture content had a direct positive effect on NO_2_^−^ + NO_3_^−^ (path coeff. = 0.39; *P* = 0.03), NH_4_^+^ (path coeff. = 0.20; *P* < 0.001) and total carbon (path coeff. = 0.16; *P* = 0.04), while total soil carbon positively covaried with NH_4_^+^ (path coeff. = 0.34; *P* < 0.001), suggesting that both moisture and total carbon are involved in mineralisation of organic matter influencing NO_2_^−^ + NO_3_^−^ and NH_4_^+^ concentrations (Fig. 3; blue arrows). Moisture content was also found to positively drive nitrification potential (path coeff. = 0.08; *P* = 0.04), in addition to the positive relationship with pH (path coeff. = 0.11; *P* = < 0.001).

### Ratio of AOB and AOA ammonia monoxygenase (*amoA*) in relation to tree health

As our SEM model identified association between tree health and the abundance of AOB, but not AOA, we further investigated the relationship between AOB:AOA ratio in association with tree health at each site. Across sites, *amoA* AOA, and AOB gene abundance varied (*P* < 0.001), but the AOB:AOA ratio was not significantly different according to tree health (*P* = 0.21) overall. Instead, the relationship between tree health and AOB:AOA ratios was site-specific. Attingham had a significantly higher ratio of AOB:AOA (93%) than symptomatic trees (63%; *P* = 0.007). In general, Winding Wood, Langdale, Great Monks Wood and Attingham had the highest ratio of AOB:AOA, while at Bigwood, Chestnuts Wood and Speculation a higher ratio of AOA:AOB was found (Fig. S2). As shown by the SEM analysis, we found that an increase in pH significantly increased the ratio of AOB:AOA (*P* = 0.007), particularly at Bigwood, where a soil pH >5.8 was found to increase AOB from less than 24% to greater than 87%. Similarly, at Chestnuts Wood, soil pH >5.8 increased AOB from 34% to greater than 73% (*P* = 0.004). While NH_4_^+^ concentration did not have an effect independent of sites, a lower concentration of NH_4_^+^ at Bigwood, Speculation and Chestnuts Wood resulted in significantly fewer AOB than AOA (*P* = 0.001).

### Microbial community composition

In soils supporting asymptomatic trees, Proteobacteria (41%), Acidobacteria (34%) and Actinobacteria (20%) were the most abundant representing 95% of the total bacterial community (data not shown). A similar pattern was observed in soils supporting symptomatic trees, where Proteobacteria (40%), Acidobacteria (37%) and Actinobacteria (17%) were most abundant representing 94% of the total bacterial community. In soils of both symptomatic trees and asymptomatic trees, Nitrospirae (a phylum containing nitrite oxidising bacteria) made up 0.02% of the total bacterial community. Within the bacterial communities, five genera, associated with nitrification (*Nitrospira, Nitrobacter, Nitrococcus, Nitrosococcus, Nitrosospira)* were found in soils of both asymptomatic and symptomatic trees. Together these genera represented 0.8% of the total bacterial composition and did not differ in diversity or relative abundance according to tree health status.

No significant differences in the composition of archaeal phyla were observed between soils of symptomatic or asymptomatic trees and were dominated by Thaumarchaeota and Crenarchaeota (between 57–58% and 25–27%). Across all sites, nitrogen cycling archaea were found in soils supporting both symptomatic and asymptomatic trees including: Nitrososphaerales (56–57%), Thermoprotei (25–26%), Thermoplasmata (6–7%) and Woesearchaeota Incertae Sedis AR16 (4–5%) (data not shown). Nitrososphaera (57%) and Nitrosopumilus (0.8%) that are involved in nitrification were also found in soils underlying both asymptomatic and symptomatic trees. Interestingly, a pronounced difference in relative abundance according to tree health status was evident with the archaeal class Methanomicrobia (Phylum Euryarchaeota), albeit forming a small component of the archaeal population comprising 3% in soils supporting asymptomatic trees and only 0.06% in soils surrounding symptomatic trees.

### Ammonia monoxygenase (*amoA*) gene composition

**Fig. 4:**
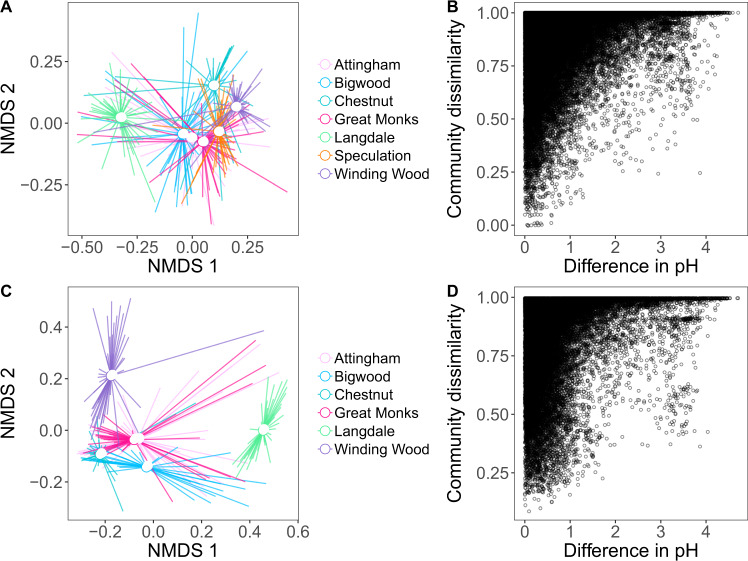
Community composition of ammonia-oxidising and nitrous oxide reducing bacteria across sites and soil pH. Differences in community composition of ammonia-oxidising bacteria (*amoA*) and nitrous oxide reducing bacteria (*nosZ*) across sites (**A**, **C**, respectively), and relationships between community dissimilarity and differences in soil pH (**B**, **D**, respectively). Distances were calculated on rarefied OTU tables using the Bray–Curtis dissimilarity index. In NMDS plots (**A**, **C**), points closer together indicate more similar communities, and hollow points show site-level centroids (mean axis values for each site). In **B**, **D**, points represent pairwise comparisons between rarefied communities, where 0 indicates two identical communities, whilst 1 indicates two communities with no common OTUs.

Following sequencing and quality filtering, 265 *amoA* bacterial OTUs were obtained, of which 150 were associated with asymptomatic trees and 157 associated with symptomatic trees, although not exclusively. Using Bray–Curtis distance, the OTU clusters showed distinct patterns based on site but not by tree health (Fig. 4A) and revealed that Langdale in particular contained a different composition of AOB compared to the other six sites. This result was supported by multivariate abundance analysis of the *amoA* bacterial gene composition across all the sites that pinpointed a significant association of *amoA* bacterial gene composition with sites (*P* = 0.001), but not with tree health status (*P* = 0.4). However, significant differences according to tree health status at the individual site and OTU level were detected. These differences occurred at only three sites and involved only two OTUs. Thus, a higher abundance of OTU106 (that had a high sequence similarity to an uncultured *amoA* bacterial clone) was found in soils associated with symptomatic trees at Winding Wood (*P* = 0.02), while at Great Monks Wood and Speculation a significantly higher abundance of OTU101 (*P* = 0.006) that also had a high sequence similarity to an uncultured *amoA* bacterial clone was found. Further multivariate abundance analyses across sites including soil variables were performed to investigate relationships with abiotic factors. Plotting the community dissimilarities against the difference in pH, we showed that differences in pH were associated with broad level shifts in community composition (Fig. 4B).

Sequencing of archaeal *amoA* genes identified 55 OTUs. Across sites, asymptomatic trees were associated with 41 OTUs, while symptomatic trees were associated with 43 OTUs. As with the AOB, there were clear site differences in community composition (*P* = 0.009), but no changes according to tree health status (*P* = 0.74). No archaeal *amoA* sequences were obtained from Winding Wood and Attingham, and this result was supported by a low abundance of *amoA* AOA found by qPCR analysis at these sites.

### Denitrifier gene composition

Following filtering, 1135 nitrous oxide reductase *(nosZ)* OTUs and nitrite reductase (*nirS*) 146 OTUs were obtained across the seven woods. Permanova analyses revealed that neither *nosZ*, nor *nirS* communities, to have distinct compositions between symptomatic and asymptomatic trees. Instead, spatial differences between sites and soil pH explained *nosZ* community composition (Fig. 4C, *P* < 0.01), whereas only site differences were observed for *nirS* communities (*P* < 0.02). For *nirS* communities, none of the measured soil parameters showed any relationship with community composition, suggesting spatial variables and other, unobserved soil variables drive *nirS* composition.

### Phylogenetic analysis

Phylogenetic analysis of the AOB *amoA* gene revealed that *Nitrosospira* sp. dominated AOB *amoA* gene libraries across sites (Fig. S3). One OTU (OTU50) grouped with *N. briensis* (98% bootstrap support), while the remaining OTUs grouped with uncultured soil bacterium clones (*amoA*) gene. The majority of OTUs clustered with *Nitrosotalea* sp. across sites. OTU1 grouped with *N. devanaterra* (97% bootstrap support) and *Nitrosopumilus martimus* was closely related to OTU 43 and OTU 53. A large portion of OTUs recovered aligned to undescribed or uncultured Thaumarchaeote and Crenarchaeote clones (Fig. S4).

Analysis of the denitrifiers and nitrite reductase (*nirS*) gene sequencing identified two OTUs (OTU 43 and OTU 30) that grouped with *Pseudomonas* sp. with 97% bootstrap support. Whilst other OTUs showed some relatedness to *Azospirillum* sp. and *Cupriavidus* sp. the majority of sequences recovered across sites grouped with uncultured bacterial *nirS* gene clones (Fig. S5). Similarly, analysis nitrous oxide reductase (*nosZ*) genes identified two clades related to *Bradyrhizobium* sp. (100% bootstrap support) and *Mesorhizobium* sp. with a large portion of OTUs grouping with uncultured bacterial *nosZ* gene clones (Fig. S6), including OTU1, 4, 6, 22, 27, 44, 50 that had less than 80% identity match to any reference taxa on Genbank and formed a distinct clade.

## Discussion

In this study we disentangled the interactions between AOB, AOA and denitrifying communities in soils associated with asymptomatic and symptomatic declining oak trees. This is the first study to provide a comprehensive, spatially replicated insight into the interactions between soil parameters, N-cycle microbial communities and tree health in temperate forests. Our study provides new empirical evidence that asymptomatic oak trees are associated with increased AOB *amoA* gene abundance, and that this is in turn modulated by soil conditions, notably pH.

It is well known that soil pH is an important factor in shaping ammonia oxidiser communities [28, 71, 72]; with AOB (not AOA) shown to positively correlate with soil pH [73]. Here, soil pH was found to be the key driver of AOB abundance, suggesting that soil pH has an indirect effect on tree health, by supporting AOB abundance and thus AOB-driven ammonia oxidation. However, the underlying mechanisms of soil pH in shaping the ammonia oxidiser community are complex as direct and indirect influencing factors can affect soil pH [74], including underlying geology, plant species and anthropogenic disturbances. While this study shows no direct significant relationship between tree health and pH, it instead suggests that ammonia oxidiser abundance, specifically AOB, may represent a novel indirect link between soil conditions and tree health, supporting our hypothesis that tree health and soil conditions interactively shape the soil N-cycle microbial community.

Recent studies have shown that vascular plants can modulate AOB abundance by emphasising the role of different plant types or functional attributes that impact surrounding soil microorganisms [35, 75]. Our observed differences in the abundance of AOB between symptomatic and asymptomatic trees may be due to the effect the tree is having on the surrounding soil environment. Healthy trees that support larger canopies produce greater levels of litter, have lower soil temperatures, and higher organic matter than declining trees. Therefore, a larger canopy from healthy trees may lead to improved soil moisture conditions and mineralisation rates, supporting higher pH ranges and by extension, AOB abundance [35, 76, 77]. Additionally, tree bark pH may also influence the pH of soil in close proximity to the trunk via stemflow [78, 79].

AOB were dominant at Winding Wood, Great Monks Wood, Attingham and Langdale, whilst AOA were dominant at Speculation, Bigwood and Chestnut. No relationship was found between AOA abundance and tree health, nor was there any difference in AOB:AOA ratios between symptomatic and asymptomatic trees. Instead, AOA abundance was driven by lower NH_4_^+^ concentrations across sites, further supporting the role of NH_4_^+^ concentration in niche differentiation of AOA and AOB [29, 32, 34]. The finding that AOB and AOA abundances were predominantly linked to different environmental factors (pH and NH_4_^+^ concentration, respectively) also explains the lack of association between tree health status and AOB:AOA ratios. Whilst tree health status may indirectly influence certain local soil conditions (e.g. pH, as discussed above), others may vary according to larger spatial-scale factors such as underlying geology or soil type, supported by our finding that NH_4_^+^ concentrations differed by site rather than tree health. Consequently, tree health would be unlikely to influence the AOB:AOA ratio as these two clades respond to different environmental axes, that themselves are differentially influenced by tree health.

It is suggested that soil depth, water-soluble carbon, and nitrate have greater effects on denitrifying community composition (*nirK, nirS* and *nosZ*) than soil pH [80]. In our study, we observed that across sites C:N ratio was driving the abundance of denitrifying genes (e.g. *nirS/nirK*), which although highly positively covaried with each other, we found no direct link between *nirS/nirK* or *nosZ* gene abundance and tree health. The lack of relationship between tree health and denitrifier abundance could be partly explained by the comparatively higher phylogenetic diversity encompassed by these groups in comparison to ammonia oxidisers, suggesting a greater array of environmental drivers.

Our study detected no compositional differences in ammonia oxidising, or denitrifying communities between symptomatic and asymptomatic trees, in contrast to our original hypothesis. Furthermore, the ability of soil parameters to explain community composition also varied strongly between the different functional groups analysed. Asymptomatic trees were found to harbour a greater abundance of AOB, yet no change in community composition was found, suggesting that any links between soil parameters and tree health status do not select for specific AOB. Only slight shifts in the abundances of specific AOB OTUs in certain sites were found, suggesting that compositional changes between trees are subtle and not generalisable.

Additionally, we showed that differences in soil pH across sites influenced AOB and *nosZ* community composition. These findings show that although composition of N-cycle communities are site-specific and likely driven by site edaphic factors, the abundance of AOB is driven by soil pH at the tree level, which may be influencing soil N-cycling, and in turn, tree health. Whilst soil pH is one of the strongest predictors of microbial community composition at the macroscale, including field scale [81, 82], studies suggest that soil pH may not be the main factor for determining composition of soil N-cycle microbial communities at the microscale level, given that pH ranges at this level may span <1 pH unit [83, 84]. Across our sites, soil pH ranged from pH 3–8 and changes in soil pH across sites were the most important factors in explaining the broad shifts in AOB composition.

Tree species, tree age and site edaphic properties can all have a significant effect on soil bacterial community composition [37, 85, 86]. Across sites, the major soil groups were classified as either surface water gleys or brown soil. However, there is likely fine scale variation within sites affecting detectable bacterial composition changes in relation to health status. Plant species have also been shown to influence the composition of denitrifiers [87–89] and modulate their surrounding soil microbiome. As the sites used in this study were mostly mixed woodlands and of varying ages, it is possible that the diversity of (spatially) closely associated trees and shrubs may have also masked any composition relationships that are occurring in response to oak tree health. Additionally, variation in oak tree density and mean basal diameter across the sites may also contribute to differences in N-cycle composition within and across sites. Given the complexity of forest microbial communities and high degree of heterogeneity in plant functional diversity and soil chemistry, detectable changes in N-cycle community composition in response to tree health status were not found within these sites. Future studies would gain from discerning whether bark pH and stemflow pH (and by extension the surrounding soil pH) are linked to oak tree health status, or whether changes in soil pH are due to secondary influences, driven by soil carbon and nitrogen as found in this study.

## Conclusion

In this study we showed that tree health status was associated with an increased abundance of AOB but had no consistent effect on community composition of N-cycling microbiota. The positive association between increased AOB abundance with asymptomatic trees and increased pH indicates that soil pH is having an indirect effect on tree health by potentially affecting AOB-driven ammonia oxidation processes. This study also shows that soil pH is being driven by C:N ratio. We propose that healthy trees that support larger canopies produce greater levels of litter. Greater leaf litter may alter the surrounding soil C:N ratio, which in turn, influences the surrounding soil pH. These findings therefore suggest that amelioration of soil acidification by increasing soil organic matter may be one management option to alleviate the stress on declining oak trees.

## Supplementary information

Supplementary Fig and Table legends

Table S1.

Fig S1.

Fig S2.

Fig S3.

Fig S4.

Fig S5.

Fig S6.
