# Supplementary material for: Relationships between nitrogen cycling microbial community abundance and composition reveal the indirect effect of soil pH on oak decline
Source: ISME J. 2020 Oct 16;15(3):623–35. doi: 10.1038/s41396-020-00801-0 (PMC8027100; doi:10.1038/s41396-020-00801-0)
Supplement: Supplementary file 1 — Supplementary Fig and Table legends [file 41396_2020_801_MOESM1_ESM.docx]

**Fig S1.** Abundance of nitrate reductase (*nirS* and *nirK*) and nitrous oxide reductase (*nosZ*) gene copies in relation to symptomatic and asymptomatic oak trees across seven UK woodlands. Data are medians with upper and lower quartile (n= 10), dots are outliers.

**Fig S2.** Ratio of ammonia monooxidase (*amoA*) AOB to AOA gene abundance. Error bars indicate the median, and upper and lower quartile derived from the least squared means (lsm).

**Fig S3.** Neighbour-joining phylogenetic tree based on bacterial *amoA* gene sequences (Fifty most abundant). The evolutionary distances were computed using the Jukes– Cantor method and are in the units of the number of base substitutions per site. Bootstrap values (based on 1000 replicates) are indicated on branch nodes. Sequences obtained in this study are shown with “OTU-” in the names. Other sequences were obtained from GenBank. The tree was out-grouped with *Nitrosococcus mobilis* (AJ298701).

**Fig S4.** Neighbour-joining phylogenetic tree based on archaeal *amoA* gene sequences (Fifty most abundant). The evolutionary distances were computed using the Jukes– Cantor method and are in the units of the number of base substitutions per site. Bootstrap values (based on 1000 replicates) are indicated on branch nodes. Sequences obtained in this study are shown with “OTU-” in the names. Other sequences were obtained from GenBank. The tree was out-grouped with *Nitrosophaera viennensis* (FR773159.1).

**Fig S5.** Neighbour-joining phylogenetic tree based on nitrite reductase (*nirS*) gene sequences (fifty most abundant). The evolutionary distances were computed using the Jukes– Cantor method and are in the units of the number of base substitutions per site. Bootstrap values (based on 1000 replicates) are indicated on branch nodes. Sequences obtained in this study are shown with “OTU-” in the names. Other sequences were obtained from GenBank. The tree was out-grouped with *Burkholderia cepacia* (AB092344.2).

**Fig S6.** Neighbour-joining phylogenetic tree based on nitrous oxide reductase (*nosZ*) gene sequences (fifty most abundant). The evolutionary distances were computed using the Jukes– Cantor method and are in the units of the number of base substitutions per site. Bootstrap values (based on 1000 replicates) are indicated on branch nodes. Sequences obtained in this study are shown with “OTU-” in the names. Other sequences were obtained from GenBank. The tree was out-grouped with *Pseudomonus aeruginosa* (X65277.1).
