# Supplementary material for: Relationships between nitrogen cycling microbial community abundance and composition reveal the indirect effect of soil pH on oak decline
Source: ISME J. 2020 Oct 16;15(3):623–35. doi: 10.1038/s41396-020-00801-0 (PMC8027100; doi:10.1038/s41396-020-00801-0)
Supplement: Supplementary file 2 — Table S1. [file 41396_2020_801_MOESM2_ESM.docx]

| **Site** | **Density**  **(oak trees /ha)** | **Diameter (cm)^a^** | **Standard Deviation (cm)** | **Mean Basel Area (m^2^ / ha)** | **Soilscapes^b^** | **Soil Series^b^** | **Major Soil Group^b^** | **Additional Soil Series^b^** | **Additional major soil group^b^** |
| --- | --- | --- | --- | --- | --- | --- | --- | --- | --- |
| Attingham | 43.15  (78.5 including recent planting) | 98 | 23.6 | 32.55* | Slowly permeable seasonally wet slightly acid but base rich loamy and clayey soils | Salop - 711m | Surface water gleys |  |  |
| Bigwood | 48.78 | 54.9 | 8.05 | 11.55 | Slowly permeable with seasonal waterlogging | Beccles 1 - 711r | Surface water gleys | Newport 4-551g | Brown soil |
| Chestnuts | 214.29 | 36.4 | 6.53 | 22.30* | Freely draining slightly acid loamy soils | Eardiston 1 - 541c | Brown soil |  |  |
| Great Monks | 43.85 | 65.8 | 13.7 | 14.91 | Slowly permeable seasonally wet slightly acid but base rich loamy and clayey soils | Oak 2 - 714c | Surface water gleys |  |  |
| Langdale | 30.95 | 72.6 | 10.7 | 12.81 | Slowly permeable seasonally wet slightly acid but base rich loamy and clayey soils | Brockhurst 1 - 711b | Surface water gleys |  |  |
| Speculation | - | 73 | 13.5 | - | Slowly permeable seasonally wet slightly acid but base rich loamy and clayey soils | Dunkeswick - 711p | Surface water gleys | Withnell 1- 611d | Podzolic soil |
| Winding Wood | 87.12 | 62.1 | 7.98 | 26.39* | Slightly acid loamy and clayey soils with impeded drainage | Hornbeam 3 - 582d | Brown soil |  |  |

**Table S1.** Study site soil classification, oak tree density and mean basal area. ^a^ Mean diameter of studied oak trees (at 1.3m tree stem height).

^b^ Based on soil classification for England and Wales [39]. *Mean basal area is at or approaching thinning threshold (between 24-27m^2^ /ha) indicating the stand is over stocked [40]. Calculated values are underestimates as they do not include the minor components due to other tree species.
