# Supplementary figures and images for: Relationships between nitrogen cycling microbial community abundance and composition reveal the indirect effect of soil pH on oak decline
Source: ISME J. 2020 Oct 16;15(3):623–35. doi: 10.1038/s41396-020-00801-0 (PMC8027100; doi:10.1038/s41396-020-00801-0)

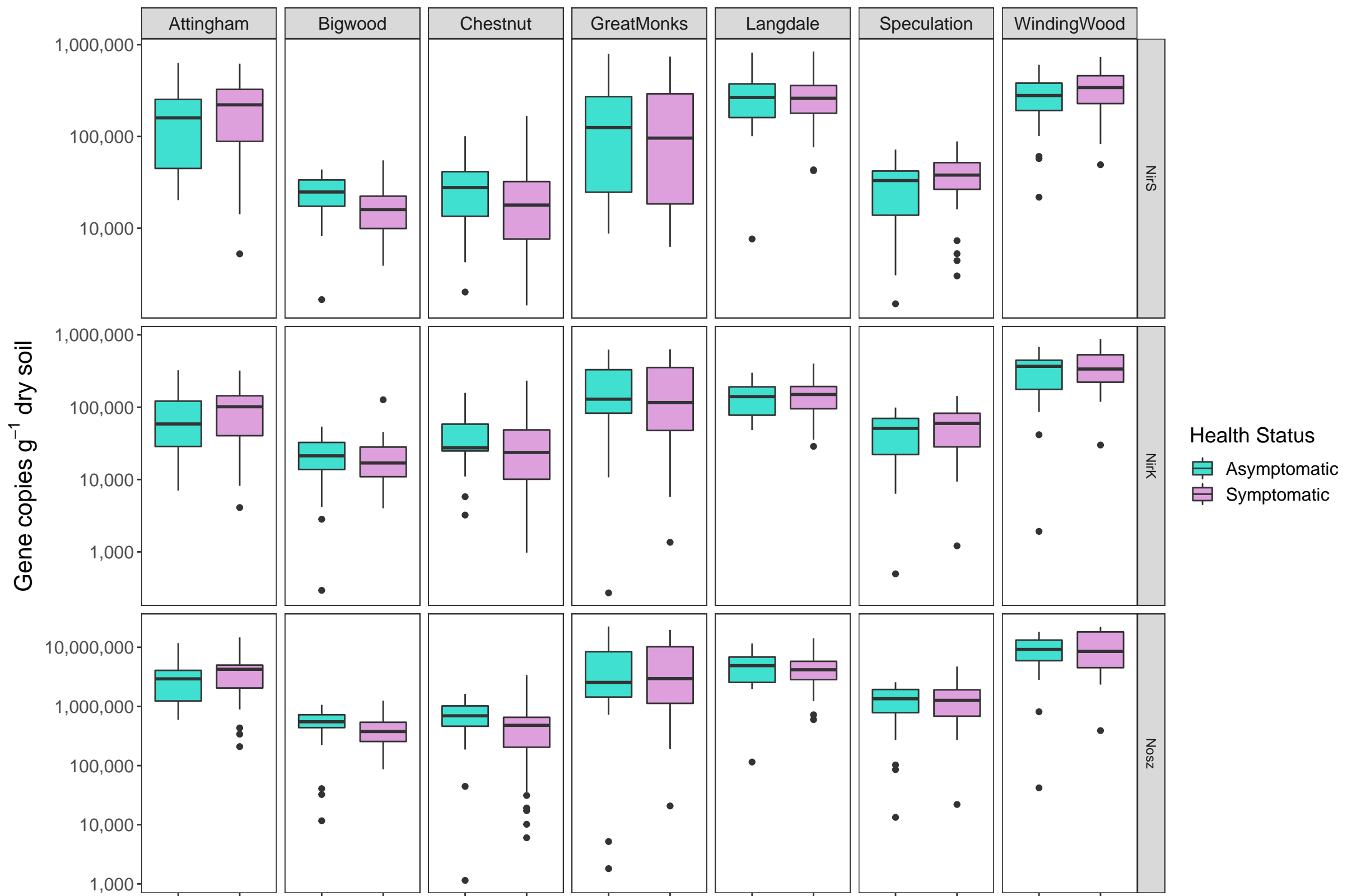

Supplement: Supplementary file 3 — Fig S1. [file 41396_2020_801_MOESM3_ESM.pdf]

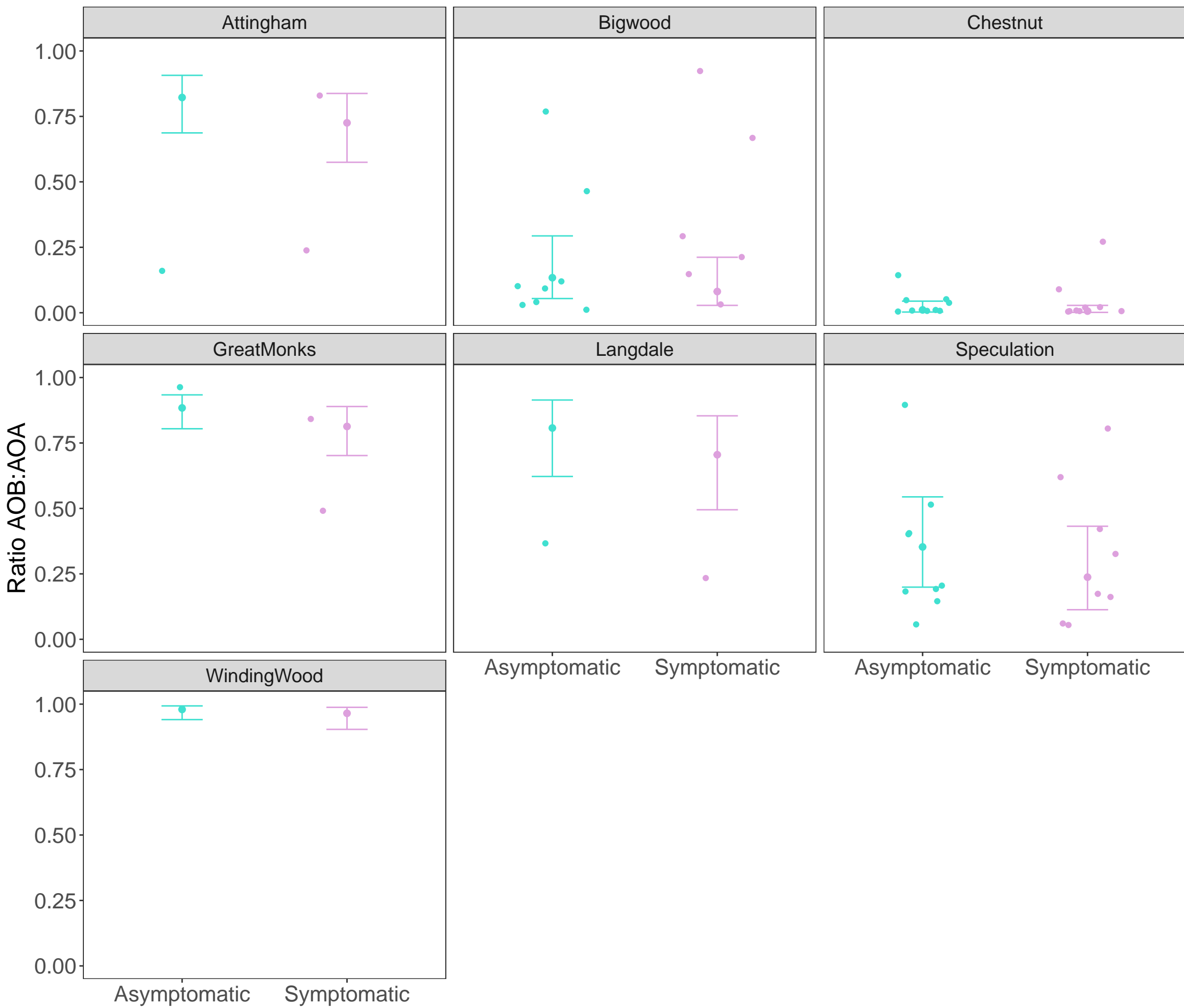

Supplement: Supplementary file 4 — Fig S2. [file 41396_2020_801_MOESM4_ESM.pdf]

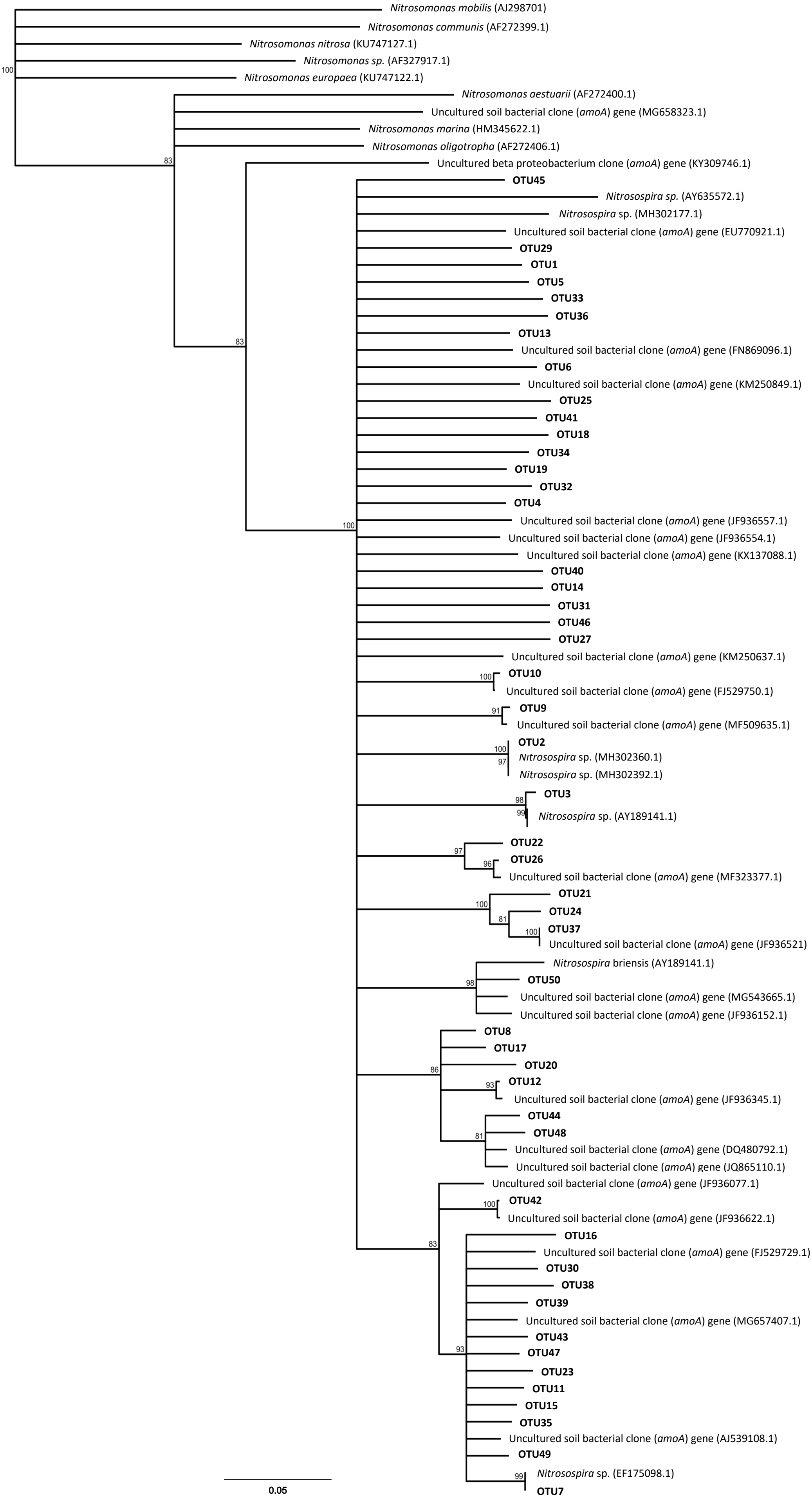

Supplement: Supplementary file 5 — Fig S3. [file 41396_2020_801_MOESM5_ESM.pdf]

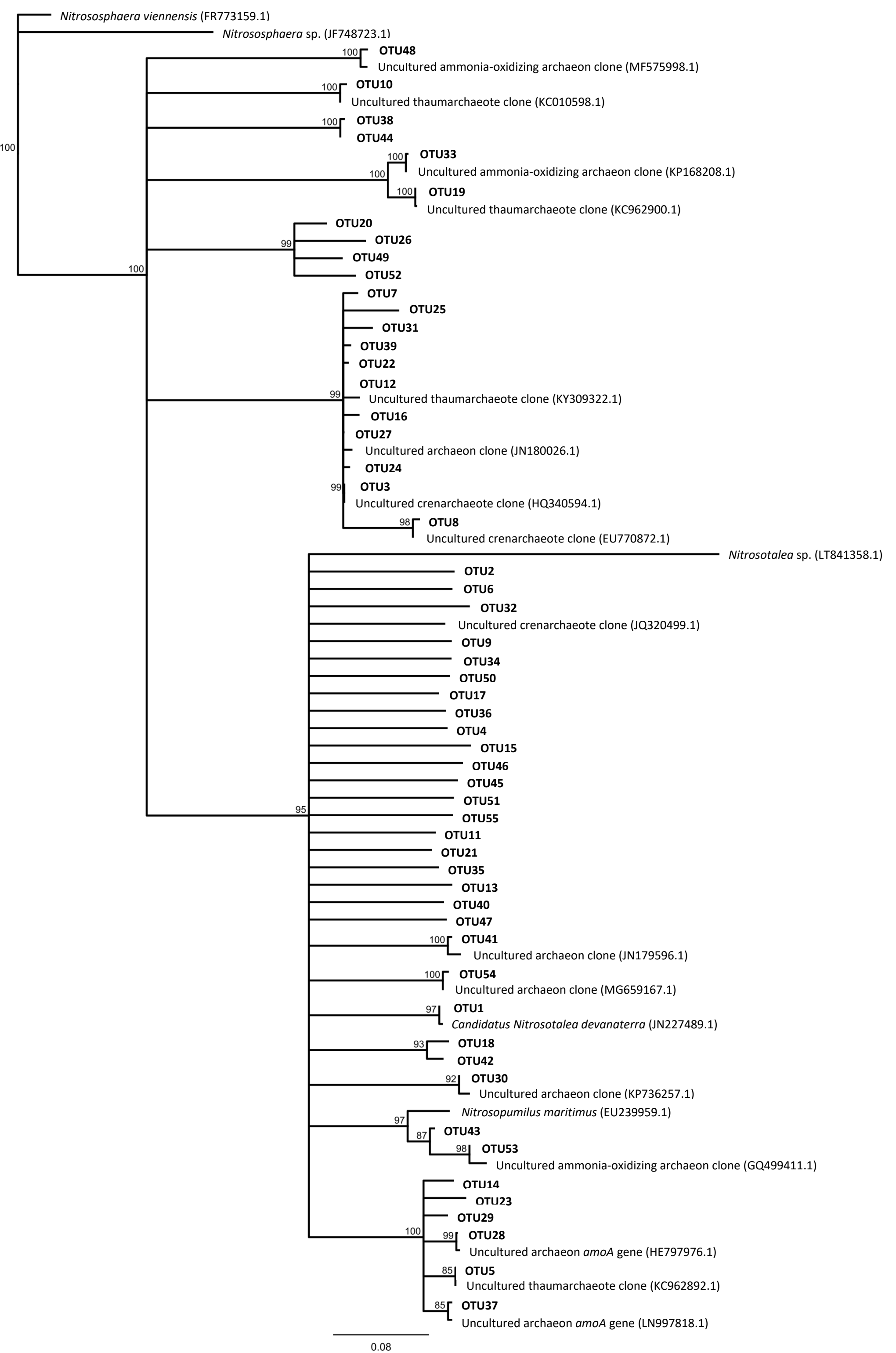

Supplement: Supplementary file 6 — Fig S4. [file 41396_2020_801_MOESM6_ESM.pdf]

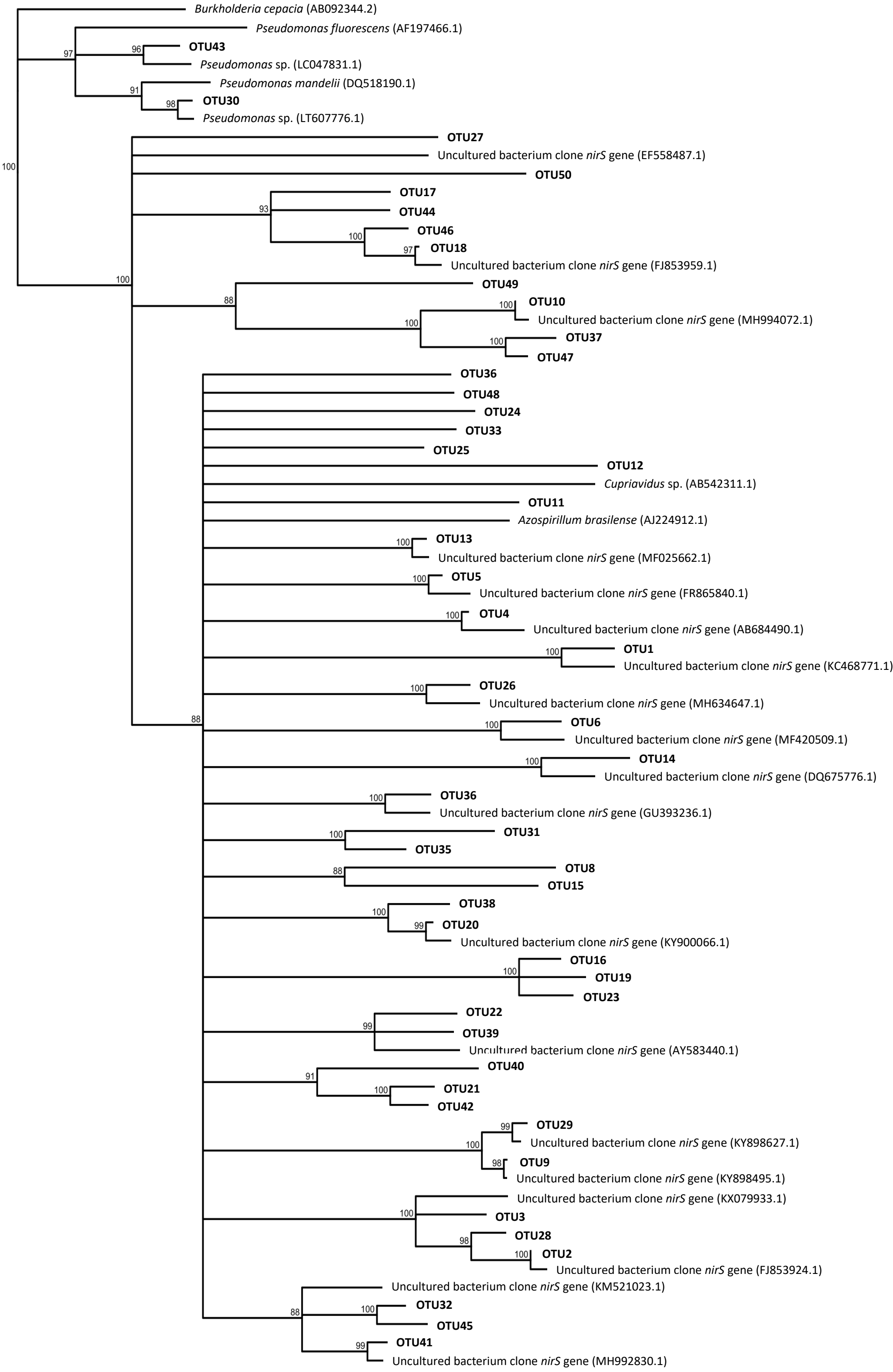

Supplement: Supplementary file 7 — Fig S5. [file 41396_2020_801_MOESM7_ESM.pdf]

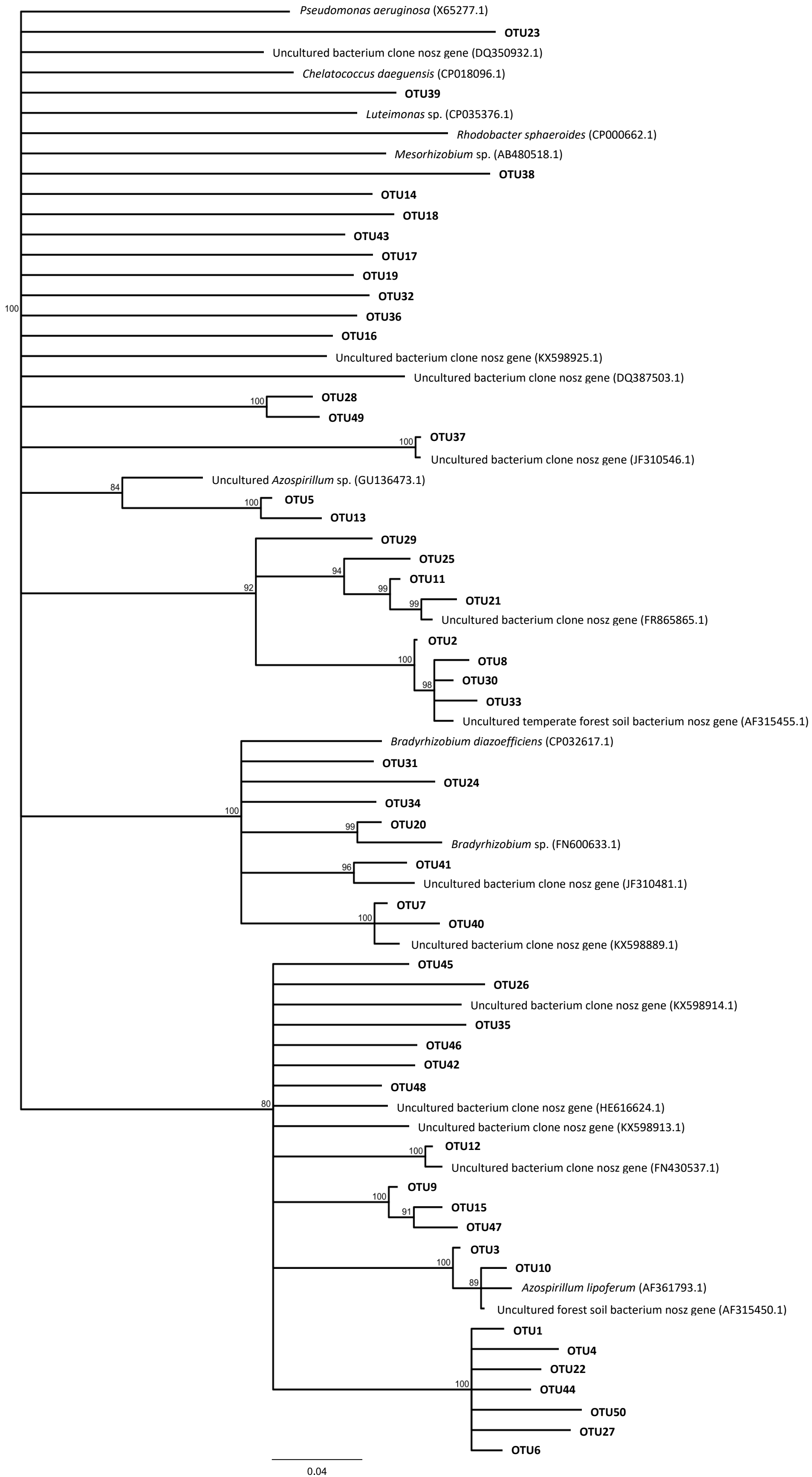

Supplement: Supplementary file 8 — Fig S6. [file 41396_2020_801_MOESM8_ESM.pdf]
